# Preserved cardiac vagal tone is associated with nocturnal glycaemic stability

**DOI:** 10.1007/s00125-026-06844-w

**Published:** 2026-09-09

**Authors:** Maria Bitsch Poulsen, Simon Lebech Cichosz, Peter Julu, Johan Røikjer, Asbjørn Mohr Drewes, Christina Brock

**Affiliations:** 1 Mech-Sense, Department of Gastroenterology, Aalborg University Hospital, Aalborg, Denmark; 2 Department of Clinical Medicine, Aalborg University, Aalborg, Denmark; 3 Department of Health Science and Technology, Aalborg University, Aalborg, Denmark; 4 The Autonomic Laboratory, William Harvey Heart Centre, Queen Mary University of London, London, UK; 5 Steno Diabetes Center North Denmark, Aalborg University Hospital, Gistrup, Denmark; 6 Department of Endocrinology, Aalborg University Hospital, Aalborg, Denmark

**Keywords:** Autonomic nervous system, Cardiac vagal tone, Continuous glucose monitoring, Glucose regulation, Glycaemic variability, Parasympathetic nervous system, Type 2 diabetes

## Abstract

**Aims/hypothesis:**

Parasympathetic dysfunction is implicated in the pathogenesis of type 2 diabetes; however, its relationship with glucose regulation remains poorly understood. Thus, the aim of this study was to investigate the association between central parasympathetic function assessed as cardiac vagal tone (CVT) and continuous glucose monitoring (CGM) outcomes during sleep in individuals with and without type 2 diabetes.

**Methods:**

This cross-sectional study included 20 individuals without diabetes and 40 individuals with type 2 diabetes, aged 45–75 years. They were home-monitored for 72 h, comprising nocturnal recordings of ECG-derived CVT measured using the ProCVT SmartSheet and Dexcom G6 CGM blinded to participants. Mean CVT was dichotomised into ‘normal’ CVT (≥5.0) and ‘poor’ CVT (<5.0). Only individuals for whom ≥70% of the total recording was classified as valid noise-free ECG data were analysed (*N*=50).

**Results:**

Across all individuals, higher nocturnal CVT predicted a lower coefficient of variation in glucose (β=−0.8, *p*=0.030) and greater time in tight range (β=5.2, *p*=0.001). In individuals without diabetes, higher CVT was associated with reduced risk of hyperglycaemia (−10.2%, *p*=0.015), time above range (−11.8%, *p*=0.015) and time in moderate hyperglycaemia (−11.8%, *p*=0.015), and increased glucose mobility (β=0.014, *p*=0.029). In all individuals, poor CVT at the 15 min segment level was associated with a 1.9% increase in glucose in the subsequent period (*p*=0.049).

**Conclusions/interpretation:**

Preserved CVT, indicating normal central parasympathetic tone, is associated with a stable and dynamically regulated glucose profile with reduced adverse glucose mobilisation during sleep. This may have implications for glycaemic regulation in individuals with autonomic neuropathy.

**Graphical Abstract:**

**Figure:**
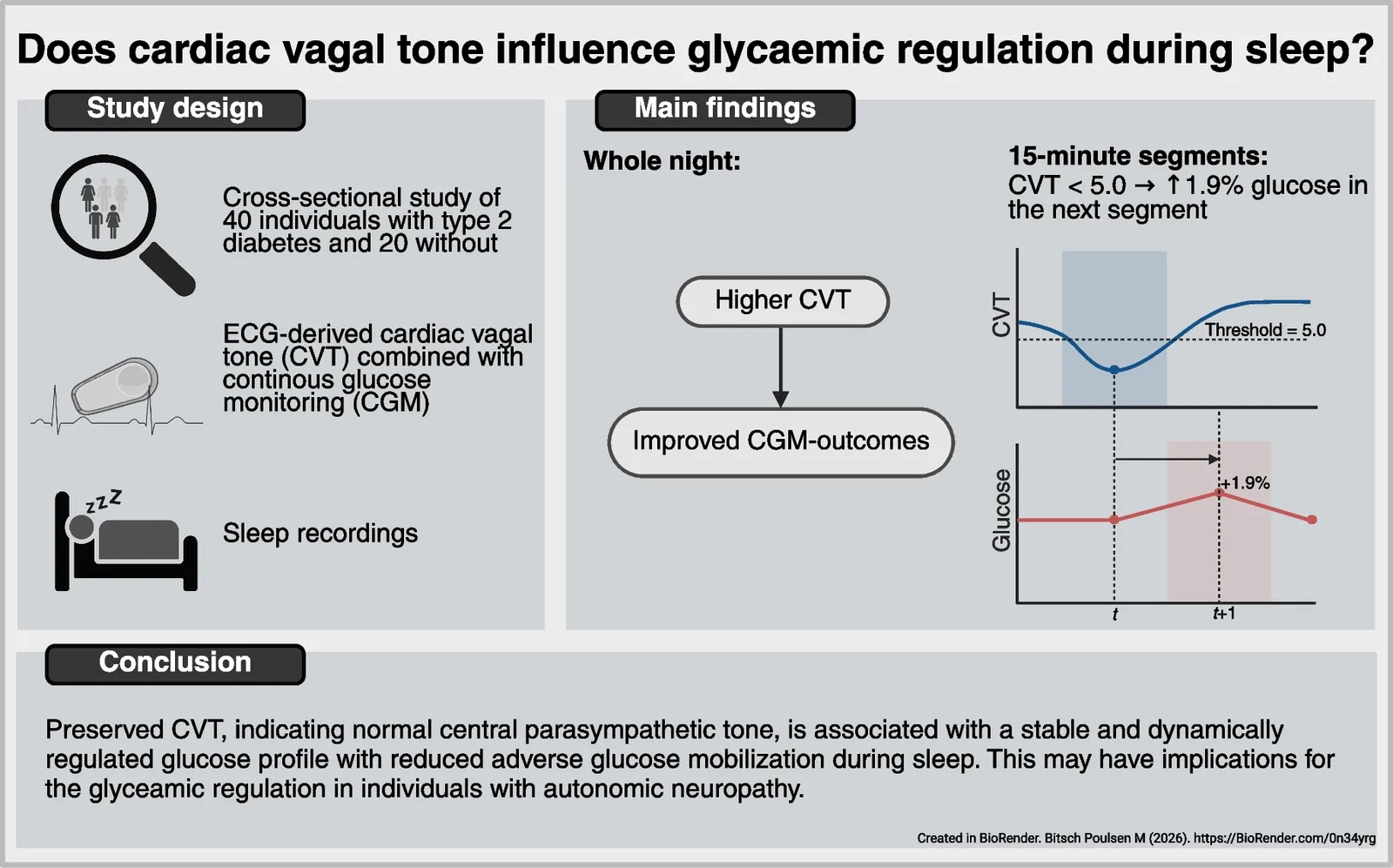

**Figure:**
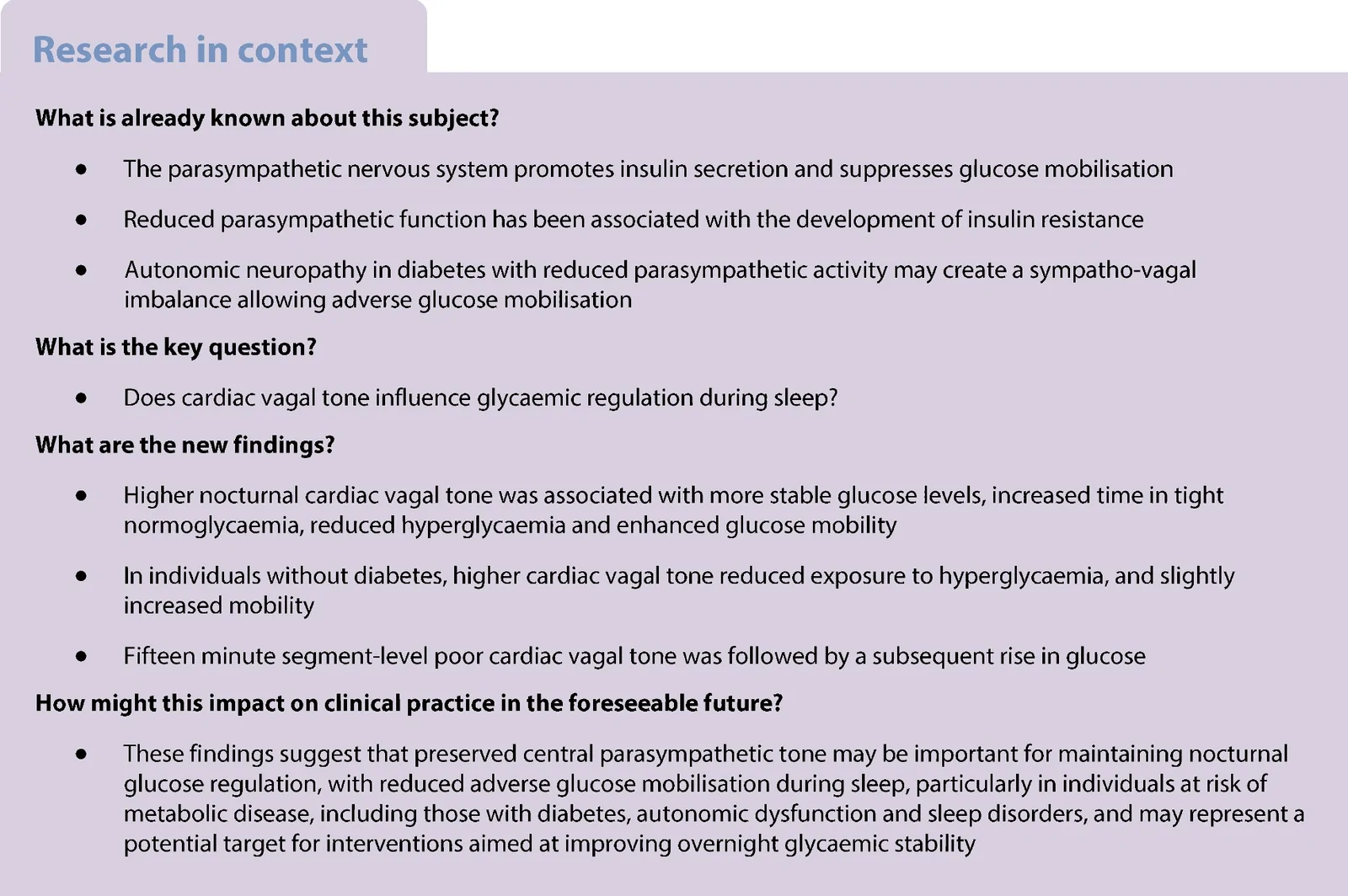

## Introduction

Type 2 diabetes is characterised by insulin resistance and progressive pancreatic beta cell dysfunction, leading to a state of persistent hyperglycaemia. In healthy individuals, glucose homeostasis is maintained through tight regulation of the hormones insulin and glucagon, coordinated by the autonomic nervous system. Parasympathetic activation promotes insulin secretion from beta cells [1, 2], while sympathetic activity can suppress insulin release and enhance glucagon secretion from pancreatic alpha cells to support glucose mobilisation in various physiological states, e.g. stress, fasting or sleep [3, 4].

Blunted parasympathetic activity has been linked to the early development of insulin resistance in both experimental models and first-degree relatives of individuals with type 2 diabetes [5, 6]. This suggests that parasympathetic dysfunction may not only be a consequence of diabetes but also a factor contributing to the pathogenesis. Type 2 diabetes is often accompanied by metabolic syndrome, a cluster of conditions that are characterised by sympathetic dominance, causing central obesity, hypertension and dyslipidaemia, in addition to insulin resistance [7]. To compensate for insulin resistance, the pancreas must increase insulin production, a demand that depends on intact parasympathetic regulation of the nervous system. However, if diabetes is complicated by autonomic neuropathy, this may lead to reduced parasympathetic activity with preserved sympathetic function [8]. This creates sympatho-vagal imbalance in favour of the sympathetic system. Consequently, individuals with type 2 diabetes may be exposed to a dual autonomic burden: an increased need for insulin and a reduced ability to produce it.

Parasympathetic dysfunction also removes an important inhibitory restraint on the sympathetic nervous system. Such loss may permit excessive sympathetic-driven glucose mobilisation, thereby promoting hyperglycaemia. Normally, parasympathetic activity predominates during sleep and suppresses sympathetic glucose release. When this nocturnal parasympathetic dominance is blunted, sympathetic driven glucose mobilisation may persist throughout the night, exposing peripheral nerves to prolonged glucotoxic conditions and thereby increasing the risk of diabetic complications. The hyperglycaemic effect of unrestrained sympathetic activity is supported by experimental evidence showing that direct pharmacological stimulation of the sympathetic nervous system induces hyperglycaemia [9]. Moreover, even after insulin-induced hypoglycaemia, individuals with type 1 diabetes exhibit insufficient parasympathetic restraint, resulting in increased glucose (a physiological overshoot) after normoglycaemia has been restored [10].

The interplay between parasympathetic activity influence, glucose mobilisation and glycaemic variability remains poorly understood. To investigate this knowledge gap, we conducted a secondary analysis of previously published recordings of cardiac vagal tone (CVT), a measure of central parasympathetic activity, in combination with transcutaneous continuous glucose monitoring (CGM) [11]. This provides an opportunity to explore how nocturnal parasympathetic activity influences glycaemic variability in real-world conditions. Nocturnal glucose levels are of particular interest as they reflect a prolonged fasting state during which endogenous glucose regulation predominates, including hepatic glucose production and counter-regulatory hormonal activity, whereby dysregulation may result in adverse glucose mobilisation. The aim of this study was to investigate the association between nocturnal CVT and CGM-derived glucose metrics in individuals without diabetes compared to individuals with type 2 diabetes.

## Methods

### Design and participants

This cross-sectional study investigated 40 individuals with type 2 diabetes aged 45–75 years, with a minimum disease duration of 5 years, who were receiving stable doses of glucose-lowering and antihypertensive medication. Type 2 diabetes were defined as an HbA_1c_ ≥48 mmol/mol (≥6.5%) within the last 12 months. Exclusion criteria were BMI >35 kg/m^2^, an eGFR ≤45 ml/min per 1.73 m^2^, severe cardiovascular disease (New York Heart Association stage IV heart insufficiency or blood pressure >150/100 mmHg) or previous bariatric surgery. Participants with type 2 diabetes were recruited from research databases with individuals from both outpatient clinics and the primary care sector in the North Denmark region to obtain a heterogeneous study population representing a broad spectrum of autonomic neuropathy severity to reflect the broader clinical population. In addition, 20 individuals without diabetes who were age-matched (±5 years) to those with diabetes were included for comparison. The study included men and women of northern European descent, with sex being defined as the sex assigned at birth. The methods are described thoroughly elsewhere [11]. Briefly, medical history and information on comorbidities and prescriptions were obtained, and blood and urine samples were collected for routine biochemistry profiling. The autonomic profile was determined using standard clinical tests fully described in detail previously [11].

Participants were monitored for 72 h at home using the ProCVT SmartSheet (ProBiometrics, Tunbridge Wells, UK) during sleep. The ProCVT SmartSheet contains six capacitive non-contact ECG sensors and one temperature sensor. ECG was performed at 1000 Hz with 24-bit resolution. CVT was derived from the ECG and calculated on a beat-to-beat basis, and each value corresponds to a rolling average of the ten preceding data points. CVT is measured using a clinically validated atropine-derived linear vagal scale (LVS), where 0 is equivalent to full atropinisation, which is complete muscarinic parasympathetic blockage of the sino-atrial node [12, 13]. Recordings were processed as described previously to remove artefacts, including exclusion of abrupt heart rate changes ≥15 beats/min (suggestive of arrhythmias or signal artefacts). Only recordings with ≥70% of the total recording comprising valid, noise-free ECG data were included in the analyses [11]. Recordings with a higher percentage of noise-free ECG data were considered to be of higher signal quality. Overnight mean CVT was dichotomised into ‘poor’ and ‘normal’ function using a mean CVT ≥5.0 LVS units to represent normal. This threshold represents a preliminary cut-off for established cardiovascular autonomic neuropathy derived from sleep recordings. This threshold is higher than daytime cut-offs in the range 2.7–3.2 LVS, probably due to the physiological increase in parasympathetic activity during sleep [11, 14]. Normal daytime levels range from 5 to 15 LVS units [15].

In addition, the participants were also monitored using transcutaneous CGM (Dexcom G6; Dexcom, San Diego, CA, USA), which were blinded to participants. It records the interstitial glucose levels every 5 min. CGM measurements were synchronised to the ProCVT SmartSheet timestamps. Table 1 lists the CGM outcomes used in the study [16, 17].

**Table 1:** Description of CGM outcomes

Category	Metric	Description
Normoglycaemia	Time in range (%)	3.9–10 mmol/l
	Time in tight range (%)	3.9–7.8 mmol/l
Hyperglycaemia	Time above range (%)	>10 mmol/l
	Time in moderate hyperglycaemia (%)	10.1–13.9 mmol/l
	Time in severe hyperglycaemia (%)	>13.9 mmol/l
Hypoglycaemia	Time below range (%)	<3.9 mmol/l
	Time in moderate hypoglycaemia (%)	3.0–3.8 mmol/l
	Time in severe hypoglycaemia (%)	<3.0 mmol/l
	Number of hypoglycaemic episodes	A hypoglycaemic episode is defined as glucose <3.9 mmol/l for at least 15 min
Risk indexes	Glycaemic risk index (%)	Overall risk of both hypo- and hyperglycaemia
	Glycaemic risk index for hyperglycaemia (%)	Risk of hyperglycaemic episodes
	Glycaemic risk index for hypoglycaemia (%)	Risk of hypoglycaemic episodes
Variability	Coefficient of variation (%)	SD normalised by the mean
	Mean amplitude of glycaemic excursions	Average magnitude of glucose fluctuations that exceed one SD from the mean
	1 h continuous overall net glycaemic action	1 h glucose variability
	Distance travelled per minute	Rate of glucose variability calculated as the sum of absolute glucose changes normalised by monitoring time
	Mobility	Variance of the differences between consecutive glucose readings relative to total glucose variance

### Ethics approval

This study was approved by the Medical Research Ethics Committee (no. 2209561) and the Danish Medicines Agency (no. 2022072376), and informed consent was obtained from the participants. This study was conducted in accordance with the Declaration of Helsinki and good clinical practice guidelines, with regular monitoring by the local independent good clinical practice unit.

### Statistical analysis

All statistical analyses were performed in R (version 4.5.1; R Foundation for Statistical Computing, Vienna, Austria). Descriptive statistics are presented as mean ± SD or median (IQR), as appropriate. Normality was assessed using QQ plots. Continuous variables were compared between groups using non-paired *t* tests or Mann–Whitney *U* tests, and categorical variables were compared using χ^2^ tests. Linear regression models or Spearman's rank correlation were used to assess the relationship between overnight mean CVT and CGM outcomes for the night with the highest mean CVT. The distribution of residuals was assessed using QQ plots.

Linear mixed-effects models (LLMs) with a random intercept for each individual were fitted to assess the effect of mean CVT on CGM metrics outcomes. The study groups were analysed separately to enable detection of group-specific patterns. Individuals with type 2 diabetes were also stratified into those with poor or normal CVT under the hypothesis that a certain level of parasympathetic activity may be required to maintain optimal glucose regulation. To further explore temporal dynamics, 15 min segments were generated to assess short-term associations between dichotomised CVT and subsequent glucose levels, and analysed using LMMs with individuals as a random intercept and adjustment for diabetes status. For the LMMs, assumptions were assessed visually using residual vs fitted plots and QQ plots. Outcomes were logarithmically transformed using the natural logarithm (log_e_) when necessary, and the relative percentage change was calculated as exp(β) − 1 for logarithm-transformed outcomes. A two-sided *p* value <0.05 was considered significant. As the present study constitutes a secondary analysis of previously collected data, no sample size calculation was performed for the current analysis, and multivariable modelling was therefore limited due to sample size considerations and the exploratory nature of the study.

## Results

### Clinical characteristics of the participants

The clinical characteristics are outlined in Table 2. Briefly, of the 60 individuals included in this study, 50 had at least one ProCVT SmartSheet recording for which ≥70% of the total recording was classified as valid noise-free ECG data and corresponding CGM data. Among those categorised as having normal CVT, participants were evenly split between individuals without diabetes and individuals with type 2 diabetes. In contrast, those categorised with poor CVT were primarily individuals with type 2 diabetes. The two groups were similar in age, sex distribution, BMI and duration of type 2 diabetes where applicable. Oral glucose-lowering treatment was the most frequent therapeutic intervention among individuals with type 2 diabetes. A total of 123 ProCVT SmartSheet recordings were analysed, and recordings from the individuals with normal CVT were longer. Even though statistically significantly different, both groups had very high-quality data, but the difference was not meaningful. Those with normal CVT had lower HbA_1c_.

**Table 2:** Clinical characteristics and quality of ProCVT SmartSheet recordings

Variable	Normal CVT (*n*=32)	Poor CVT (*n*=18)	*p* value
Study group			0.032
Without diabetes	16 (50)	3 (16.7)	
Type 2 diabetes	16 (50)	15 (83.3)	
Age	61 ± 8	63 ± 7	0.577
Male	20 (62.5)	8 (44.4)	0.348
BMI	28.2 (24.5–31.7)	29.8 (25.8–31.9)	0.301
Diabetes duration	10.5 (7.0–15.5)	13.0 (7.5–22.2)	0.601
Glucose-lowering medication			0.610
Non-insulin treatment	10 (31.3)	10 (55.6)	
Insulin treatment	2 (6.3)	0 (0)	
Combination (insulin + non-insulin)	4 (12.5)	5 (27.8)	
ProCVT SmartSheet recordings			
Recordings	71	52	
Recording length (h:mm)	07:49 ± 01:24	06:37 ± 01:53	<0.001
High-quality data^a^ (%)	98.9 ± 1.1	99.6 ± 0.4	<0.001
CVT	8.7 (6.7–10.2)	4.2 (3.3–4.4)	<0.001
Glucose metrics			
HbA_1c_ (mmol/mol)	42 (35–52)	52 (48–55)	0.033
HbA_1c_ (%)	5.9 (5.3–7.0)	7.0 (6.6–7.2)	0.033
Glucose (mmol/l)^b^	7.3 (6.4–9.4)	9.0 (8.1–9.6)	0.074
Time in range (%)^b^	90 (68–99)	72 (62–81)	0.082
Coefficient of variation (%)^b^	19.2 (14.6–24.9)	23.6 (18.7–26.0)	0.063

Data are *n* or *n* (%) for categorical variables and mean ± SD or median (IQR) for continuous variables

^a^The quality of each datapoint is rated on a scale from 1 to 15, with 15 being the highest. A score ≥13 indicates high-quality data

^b^Based on the entire 72 h monitoring period

### Effect of mean CVT on nocturnal CGM outcomes

**Fig. 1:**
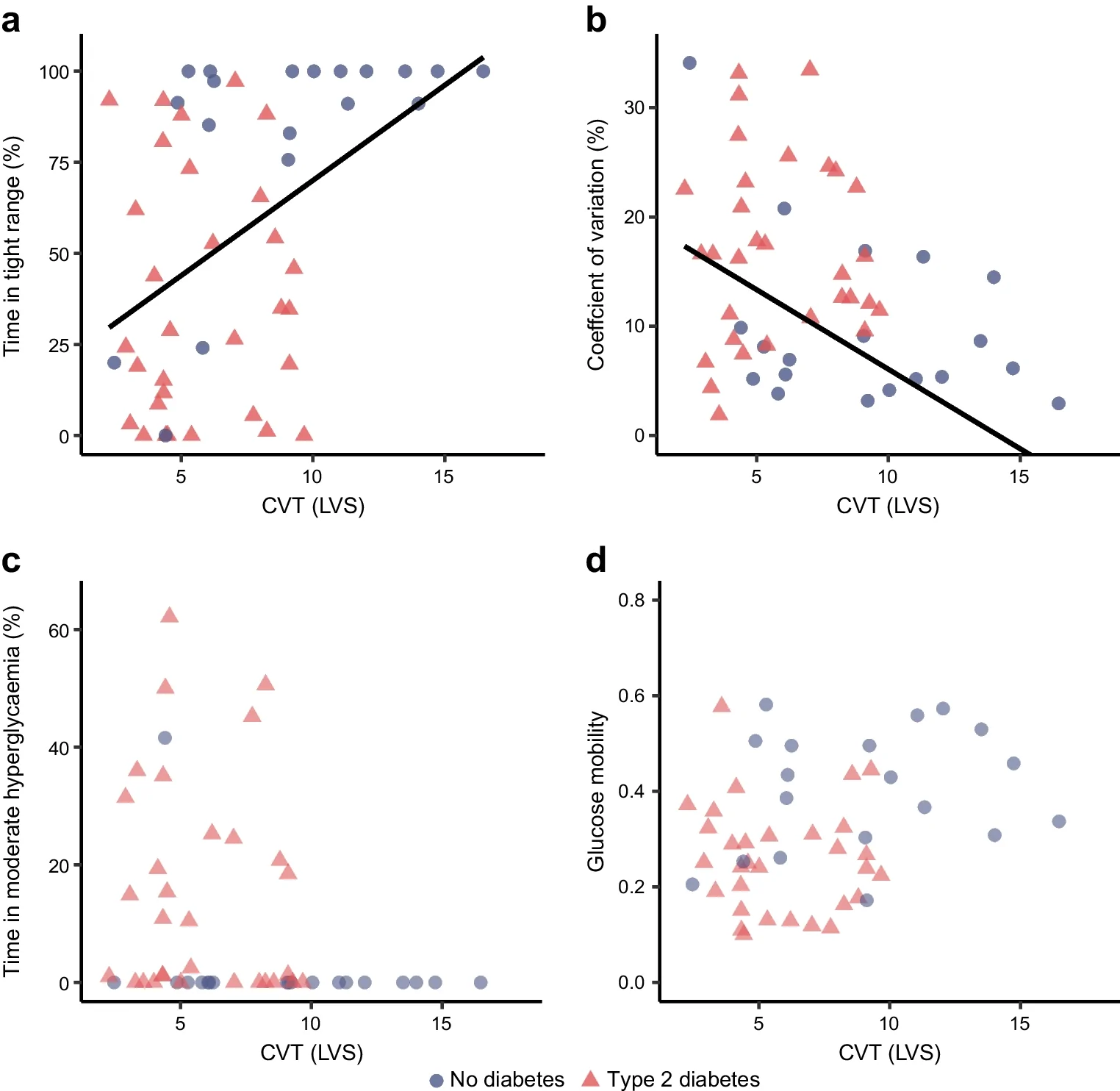
Scatterplots of the association between overnight mean CVT across both individuals with type 2 diabetes and those without, and the following CGM metrics: (**a**) time in tight normoglycaemic range (3.9–7.8 mmol/l), (**b**) the coefficient of variation, (**c**) time in moderate hyperglycaemia (3.0–3.8 mmol/l) and (**d**) glucose mobility. LVS, linear vagal scale

Linear regression analysis across all individuals revealed two distinct significant patterns: that CVT can predict the coefficient of variation (β=−0.8; 95% CI −1.4, −0.1; *R*^2^=0.10, *p*=0.030; Fig. 1b) and time in tight range (β=5.2; 95% CI 2.3, 8.1; *R*^2^=0.21, *p*=0.001; Fig. 1a). Spearman regression analysis showed a negative association between overnight mean CVT and time in moderate hyperglycaemia (ρ=−0.29; *p*=0.039; Fig. 1c) and a positive association between overnight mean CVT and glucose mobility (ρ=−0.29; *p*=0.043; Fig. 1d). Increased mobility is indicative of actively regulated glucose, characterised by frequent minor fluctuations. No other significant associations were observed between overnight mean CVT and CGM outcomes. Collectively, these results suggest that higher central parasympathetic activity promotes a stable but adaptive, moment-to-moment regulation of glucose, with more time spent within a narrow normoglycaemic range.

### Effect of mean CVT on CGM outcomes in individuals without diabetes

Analysis of the individuals without diabetes revealed that higher CVT was associated with meaningful changes in certain CGM outcomes. These are listed in Table 3. Specifically, an increase in CVT was linked to a relative reduction in general risk index for hyperglycaemia, time above range and time in moderate hyperglycaemia. Conversely, higher CVT was associated with trends towards improved glycaemic management, with more time in normoglycaemia (time in range and time in tight range). Higher CVT was associated with a small increase in mobility in the non-log-transformed outcome. Overall, these findings suggest that better CVT is linked to reduced hyperglycaemic exposure, trends towards improved glycaemic management, and slightly increased mobility in individuals without diabetes.

**Table 3:** Effect of CVT on selected CGM outcomes in the individuals without diabetes

	Model	Effect	95% CI	Type of effect	*p* value
Generalised risk of hyperglycaemia	Log LMM	−10.2%	−17.5, −2.5	Relative	0.015
Time above range	Log LMM	−11.8%	−20.1, −2.8	Relative	0.015
Time in moderate hyperglycaemia	Log LMM	−11.8%	−20.1, −2.8	Relative	0.015
Time in range	Log LMM	1.9%	0.0, 3.8	Relative	0.055
Time in tight range	Log LMM	15.5%	1.1, 32.0	Relative	0.059
Mobility	LMM	0.014	0.002, 0.025	Absolute	0.029

For logarithm-transformed outcomes, effects are shown as relative percentage changes. For mobility, the effect is an absolute change in the measured scale

Log LMM, linear mixed-effect model with log-transformed outcome

### Effect of mean CVT on CGM outcomes in individuals with type 2 diabetes

In individuals with type 2 diabetes, no significant associations between mean CVT and CGM outcomes were observed overall. However, in individuals with type 2 diabetes and normal CVT, mean CVT was positively associated with distance travelled per minute (β=0.1; 95% CI 0.02, 0.14; *p*=0.020), potentially reflecting more adaptable glucose regulation with rapid fluctuations; however, the effect was small.

### Effect of mean CVT on short-term dynamic glucose mobilisation

**Fig. 2:**
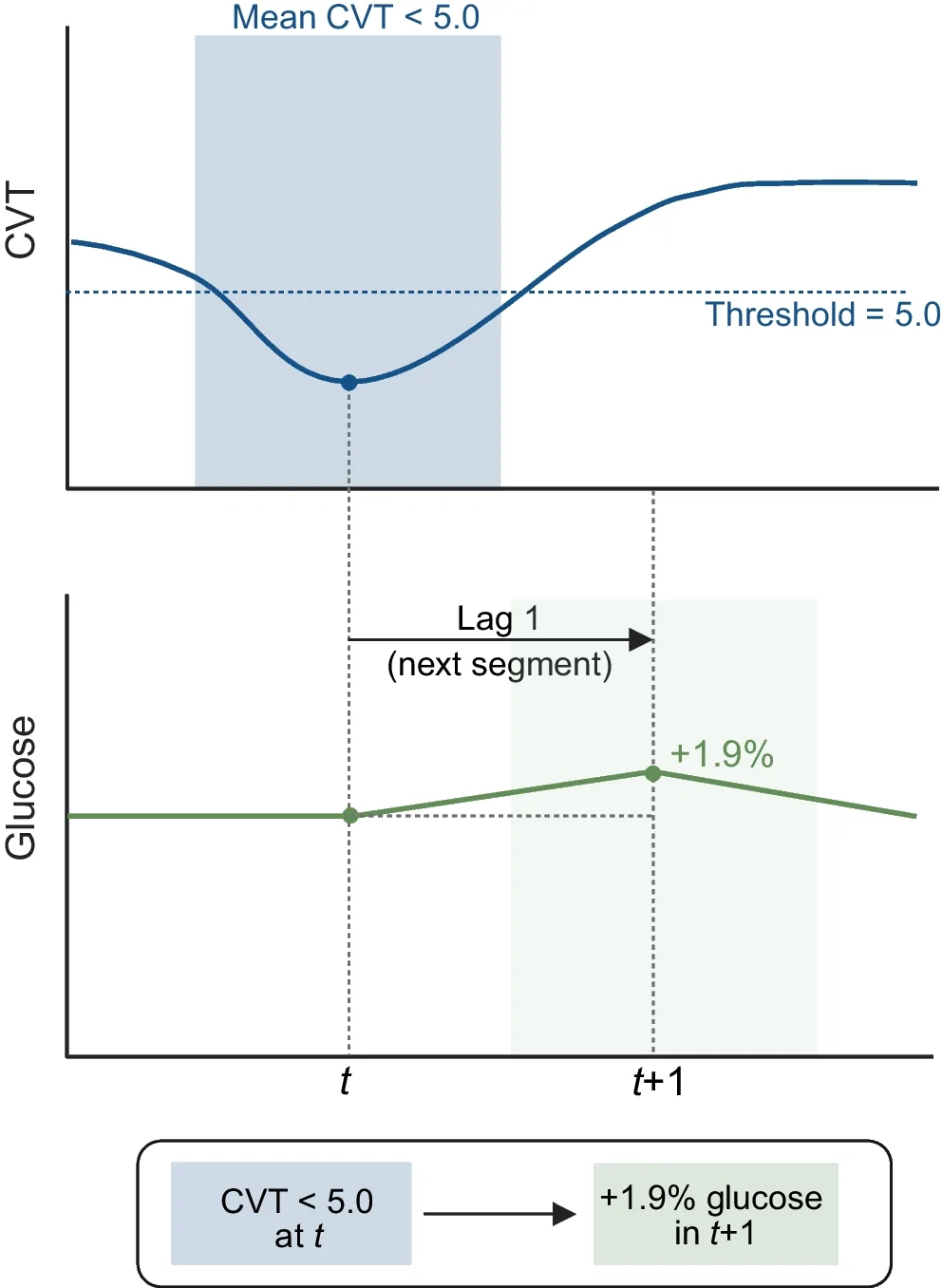
Schematic representation of the dynamic effect of CVT on glucose mobilisation. Created in BioRender. Bitsch Poulsen, M. (2026). https://BioRender.com/le0vcj1

Overnight sleep data were divided into 15 min segments to determine whether CVT has a short-term effect on the subsequent glucose level (i.e. the next 15 min). Across all individuals, an LMM adjusted for type 2 diabetes showed that segments categorised as poor CVT were associated with a 1.9% increase in mean glucose (95% CI 0.01, 3.8; *p*=0.049) in the following segment. A schematic representation is shown in Fig. 2. This may suggest that transient reductions in parasympathetic function are followed by short-term elevations in glucose resulting from endogenous glucose mobilisation.

## Discussion

Diabetic autonomic neuropathy is a troubling complication of diabetes, but it is often overlooked. The results of the present analysis showed that higher nocturnal CVT was associated with lower glucose variability, more time in the tight normoglycaemic range, less hyperglycaemia and greater glucose mobility. In individuals without diabetes, higher CVT was particularly linked to reduced hyperglycaemia and slightly increased mobility. At the 15 min segment level, poor CVT was followed by a modest increase in glucose, suggesting an acute parasympathetic influence on glucose regulation. This may have implications for long-term metabolic risk, as increased glycaemic variability and hyperglycaemic exposure are both associated with adverse diabetes-related outcomes.

In this study, individuals with normal CVT were evenly distributed between the individuals without diabetes and individuals with type 2 diabetes, whereas those with poor CVT primarily consisted of individuals with type 2 diabetes. This reflects the fact that autonomic dysfunction is frequent in individuals with diabetes [18]. Poor CVT was associated with higher HbA_1c_, and mean glucose, time in range and coefficient of variation showed similar directional patterns, suggesting that preserved parasympathetic function may be relevant for better glycaemic management.

Although the data quality of the ProCVT SmartSheet recordings was high across groups, the recordings were shorter in those with poor CVT. This may reflect fragmented sleep or lower sleep quality in individuals with parasympathetic dysfunction. During sleep, the autonomic balance shifts to parasympathetic dominance [11, 19], and thus this is the best time to assess parasympathetic integrity. It has been shown in healthy individuals that suppression of deep non-rapid eye movement (REM) sleep or sleep fragmentation reduces glucose tolerance [20, 21], suggesting that both parasympathetic function and sleep quality are important for maintaining glucose homeostasis. Obstructive sleep apnoea is highly prevalent in individuals with type 2 diabetes, with reported rates of up to 50% [22, 23], and sleep disruption has a substantial negative impact on quality of life [24]. It has been reported that treatment with continuous positive airway pressure improves glycaemic management assessed as a lowered HbA_1c_ [25, 26], suggesting that restoring sleep quality can enhance glycaemic management, at least partially by improving parasympathetic function. This mechanism may not receive sufficient awareness in clinical practice.

### Implications of parasympathetic activity in nocturnal glycaemic regulation

Overall, the results for the combined study population demonstrated that higher central parasympathetic activity promotes a stable but adaptive, moment-to-moment regulation of glucose, with more time spent within a narrow normoglycaemic range and less time in the hyperglycaemic range. In contrast, but in support of our findings, Ohlsson et al reported increased glucose variability in individuals with abnormal expiration–inspiration ratio [27], a cardiovascular autonomic reflex test assessing parasympathetic function. This is consistent with the physiological understanding that parasympathetic activity contributes to fine-tuned metabolic homeostasis [28].

Individuals with type 2 diabetes showed only limited associations between CVT and nocturnal glycaemic regulation. A small positive effect on distance travelled per minute among those with normal CVT was shown, suggesting that type 2 diabetes and subsequent metabolic disease may attenuate or constrain the role of parasympathetic activity in nocturnal glycaemic regulation. In the individuals without diabetes, higher CVT was associated with reduced hyperglycaemic exposure, possibly indicating improved glycaemic management, and slightly increased mobility. These observations suggest that, even in the absence of metabolic disease, diminished CVT is associated with increased glucose mobilisation during sleep. Intensified insulin therapy has been shown to improve HbA_1c_ and parasympathetic function, suggesting that cardiovascular autonomic neuropathy may be at least partly reversible with improved glycaemic management [29].

In terms of the short-term effect of poor CVT on glucose, it was observed that the parasympathetic nervous system also exerts an acute influence on glucose regulation, with transient reductions in CVT followed by modest increases in glucose. This highlights the dynamic role of autonomic tone in nocturnal glycaemic regulation, and suggests that even brief parasympathetic impairments can trigger short-term glucose mobilisation. Preclinical evidence also supports a central role of the parasympathetic nervous system in glucose homeostasis, whereby cholinergic activation has been shown to reduce glucose, while anticholinergic blockade instead increases glucose, indicating that central parasympathetic pathways may modulate peripheral glucose metabolism [30].

Lindmark et al reported evidence of reduced parasympathetic activity with sympathetic dominance in individuals with low insulin sensitivity, indicated by frequency-derived measures from heart rate variability analyses, specifically a higher ratio of low frequency power to high frequency power and overall lower high frequency power [5]. Similarly, sympathetic overdrive with diminished parasympathetic activity have been observed in obese adolescents with impaired glucose regulation [31]. Acute increased sympathetic tone resulting from stress is also known to disrupt glucose control in individuals with diabetes [32, 33]. Together with our findings from the individuals without diabetes, these results suggest that autonomic dysregulation may precede and contribute to the development of dysglycaemia, rather than solely being a consequence of type 2 diabetes. Longitudinal studies including individuals with impaired glucose tolerance are therefore needed to determine whether impaired nocturnal CVT represents an early mechanistic link in this progression.

Impaired parasympathetic function can initiate a vicious cycle whereby reduced parasympathetic activity limits insulin secretion, leading to elevated blood glucose, which further stresses the autonomic system. Over time, this progressive loss of endogenous regulation may increase reliance on pharmacological treatment and the risk of long-term complications. Preserving parasympathetic function may therefore be important for short-term glycaemic management, but also for limiting the downward spiral of metabolic and autonomic deterioration.

### Technological advantages of this study

This study benefits from the combined use of CGM and long-term sleep recording of CVT, providing insights into real-world glucose regulation in a home-based environment. As dietary intake affects glucose measurements, sleep recordings provide a less biased assessment, as they are less influenced by food intake. CVT was selected as the measure of parasympathetic function because it possesses advantages over cardiovascular autonomic reflex tests, which are clinically recommended for assessment of cardiovascular autonomic neuropathy [34]. Among these recommended reflex tests, only the expiration–inspiration ratio is predominantly parasympathetically mediated [35]. Unlike single-timepoint reflex tests with active participation, nocturnal CVT provides a more integrative measure of parasympathetic function, capturing autonomic regulation during the period in which parasympathetic activity is physiologically most pronounced. Heart rate variability measures are other options, but the parasympathetic origin of such indices can be challenged [36]. A further advantage of CVT is its insensitivity to beta-blockers. Although beta-blockers artificially prolong RR intervals, the prolongation occurs relatively uniformly. As CVT is calculated using a rolling average, the extension of RR intervals does not influence CVT. Longer diabetes duration is associated with more advanced complications and may reflect overall disease severity, potentially overlapping with CVT as a marker of autonomic dysfunction. However, no difference in diabetes duration was observed between the CVT groups (Table 2), suggesting limited confounding by duration, although residual confounding by cumulative disease burden cannot be excluded. Therefore, diabetes duration was not included in the multivariable analyses. In addition, mean CVT only shows approximately 10% night-to-night fluctuations, making it difficult to assess meaningful within-individual effects on the CGM outcomes [11]. This finding may also suggest that whole-night averages capture more stable autonomic influences on glycaemic regulation, whereas shorter time segments could be more sensitive to transient physiological interactions that are not reflected in nightly mean values.

### Limitations of the study

This is an explorative cross-sectional study with small sample size; the findings are therefore preliminary. Further studies are required, preferably longitudinal studies with larger cohorts, including individuals with impaired glucose tolerance and individuals with diet-treated and pharmacologically treated type 2 diabetes, to further investigate whether impaired parasympathetic function contributes to dysglycaemia. The interpretation of autonomic effects in type 2 diabetes is further complicated by use of glucose-lowering therapies. All individuals with type 2 diabetes in this study received glucose-lowering medication, which artificially improves CGM outcomes. Therefore, the physiological effect of parasympathetic function and glucose regulation in our cohort may be underestimated. The study was not designed to investigate the potential effect of diabetes treatments such as glucagon-like peptide-1 receptor agonists or sodium–glucose co-transporter 2 inhibitors on CVT. Moreover, the CVT cut-off of 5.0 remains preliminary and requires validation in independent populations before clinical application. The short-term effect of CVT on glucose is probably limited by the lower sampling rate of the CGM monitor (every 5 min vs 1000 Hz), therefore its immediate influence on rapid glucose fluctuations may not be fully captured. Nonetheless, the ability to analyse 15 min segments showed associations between CVT and glucose levels in subsequent segments when real-time glucose mobilisation is taken into consideration. Despite these limitations, the findings in this study provide valuable insights into the role of parasympathetic tone in the modulation of glycaemic stability, and highlight avenues for future research.

### Conclusion

Preserved nocturnal CVT, reflecting intact central parasympathetic function, was associated with more stable and dynamically regulated glucose regulation, with reduced exposure to hyperglycaemia. In short-term glycaemic regulation, reductions in CVT preceded modest increases in glucose, demonstrating an acute influence of parasympathetic activity on glucose. These findings suggest that parasympathetic dysfunction may contribute to dysglycaemia and potentially promote a progressive cycle of worsening glycaemic management and autonomic impairment, accelerating the progression of autonomic neuropathy.

## Data Availability

The data supporting the conclusions of this article will be made available upon reasonable request to the corresponding author. Access will be provided in accordance with applicable ethical and legal requirements.

## Abbreviations

CGM: Continuous glucose monitoring
CVT: Cardiac vagal tone
LMM: Linear mixed-effects model
LVS: Linear vagal scale

## Authors’ relationship and activities

The authors declare that there are no relationships or activities that might bias, or be perceived to bias, their work.
